# Impact of an Altered PROX1 Expression on Clinicopathology, Prognosis and Progression in Renal Cell Carcinoma

**DOI:** 10.1371/journal.pone.0095996

**Published:** 2014-05-05

**Authors:** Tao Lv, Yanfeng Liu, Jianping Zhang, Le Xu, Yu Zhu, Hankun Yin, Huimin An, Zongming Lin, Youhua Xie, Lian Chen

**Affiliations:** 1 Department of Urology, Zhongshan Hospital, Fudan University, Shanghai, China; 2 Key Laboratory of Medical Molecular Virology (MOE & MOH), Institute of Biomedical Sciences, Shanghai Medical College, Fudan University, Shanghai, China; 3 Department of Pathology, Children Hospital, Fudan University, Shanghai, China; Vanderbilt University Medical Center, United States of America

## Abstract

The transcription factor PROX1 (prospero homeobox 1) has a critical role in the development of various organs, and has been implicated in both oncogenic and tumor-suppressive functions in human cancers. However, the role of PROX1 in the development of renal cell carcinomas (RCCs) has not yet been studied. Here, we reported that PROX1 expression was decreased in human RCC tissues compared with adjacent normal tissues. In RCC tissues, however, poorly differentiated RCC expressed higher PROX1 levels compared with well-differentiated RCC. In addition, the PROX1 immunostaining levels were positively correlated with tumor nuclear grade and lymph node metastasis. Further, high PROX1 expression indicated poor survival for patients. These findings imply that in the different developmental stages of RCC, PROX1 may exert distinct functions according to the specific microenvironment of tumor. Moreover, *in vitro* experiments revealed that PROX1 overexpression enhanced the proliferation and migration of RCC cells; conversely, PROX1 depletion by siRNA attenuated the proliferation and migration of RCC cells. Collectively, these observations suggest that PROX1 plays an important role in RCC development and progression, and PROX1 may be a novel target for prevention and treatment of RCC.

## Introduction

Renal cell carcinoma (RCC) arises primarily in the renal parenchyma and accounts for over 90% of kidney carcinomas [1]. Clear cell RCC (ccRCC) is the most frequent form of RCC, with an incidence of 75%, followed by non-ccRCC types, including papillary tumors (10%), chromophobe tumors (5%) and other rare types [2]. RCC has the highest mortality rate among genitourinary cancers and its incidence has risen steadily, with a global incidence of approximately 200 000 new cases and a mortality rate of more than 100 000 patients annually [3]. Nephrectomy is an effective treatment for localized RCC disease, but advanced RCC is still highly lethal, with a 5-yr survival of 53% [4]. Treatment of RCC is hampered by the limited understanding of the pathogenesis of RCC, particularly the lack of insight into molecular mechanisms and pathways altered during its development. Moreover, RCC is associated with an extensive and complex array of genetic defects, further complicating the clinical picture.

The homeobox gene *PROX1* is an evolutionarily conserved transcription factor that controls cell differentiation and plays essential roles during embryonic development of the lens, retina, liver, pancreas, and lymphatic vasculature [5], [6], [7], [8], [9]. Although the role of PROX1 in embryonic organogenesis and lymphatic vasculogenesis is well established, little is known about its function in adult tissues. In recent studies, both oncogenic and tumor-suppressive functions have been ascribed to PROX1 in a variety of different human cancers. PROX1 participates in the transition from benign colon adenoma to carcinoma [10], and in mouse hemangioendothelioma cells, stable overexpression of PROX1 induces an invasive phenotype and promotes expression of genes involved in cell migration [11]. On the other hand, PROX1 expression is down-regulated in pancreatic cancer tissues, and loss of PROX1 function is associated with decreased patient survival [12]. In carcinomas of the biliary system, epigenetic silencing and genomic deletions of the *PROX1* gene, and the attendant drastic reduction in PROX1 protein levels, suggest that PROX1 acts as a tumor suppressor [13]. Nevertheless, the exact mechanisms by which PROX1 regulates the differentiation and proliferation of cancer cells to influence overall prognosis are largely unknown.

PROX1 is multifunctional protein whose physiological functions may change according to developmental stage, organ, or type of cancer. Previous investigations have documented that *PROX1* mRNA is expressed in both human embryonic and adult kidney tissues [14]. A recent cancer gene profiling study revealed that *PROX1* mRNA is significantly decreased in renal cancer tissue compared to adjacent normal tissue [10]. These observations raise the question of whether a relationship exists between PROX1 and RCC, a question that has not yet been studied. Here, we investigated the expression of PROX1 in human RCC and subsequently explored the potential role played by PROX1 in the tumorigenesis and development of RCC.

## Materials and Methods

### Ethics statement

The study was approved by the Clinical Research Ethics Committee of Zhongshan Hospital of Fudan University (Shanghai, China). Written informed consent was obtained from all patients for use of those tissue samples in research.

### Patients

Fresh tissue specimens from a series of 92 RCC patients who underwent resection in 2012 at Zhongshan Hospital of Fudan University were obtained for real-time quantitative polymerase chain reaction (qPCR) and Western blot analysis. The tissue samples were snap frozen in liquid nitrogen immediately after resection and stored at −80°C until further analysis.

In addition, archived formalin-fixed and paraffin-embedded tissue specimens obtained from 115 consecutive cases of patients who had undergone radical nephrectomy or nephron-sparing surgery for unilateral, sporadic RCC in 2005 were obtained for immunohistochemistry analysis. None of the patients had received chemotherapy or radiotherapy before surgery. Tumor stage was determined according to the 2009 International Union Against Cancer TNM classification system. Tumor differentiation was graded using the Fuhrman classification system [15]. The use of samples was approved by the Ethics Committee of Zhongshan Hospital. Documented informed consent was obtained from each patient prior to participation in this study.

### Real-time qPCR

Total RNA was extracted from 92 paired RCC samples and corresponding adjacent normal tissues by TRIzol (Invitrogen, Carlsbad, CA, USA) according to the manufacturer's instructions. cDNA was synthesized from total RNA (0.5 µg) in a total reaction volume of 10 µl using the PrimeScript RT reagent Kit (TaKaRa Bio, Shiga, Japan). The reaction mixture was incubated at 37°C for 15 min, heated briefly (10 s) at 85°C, and then cooled to 4°C. The following primer pairs were used for PCR: *PROX1*, 5′-GGG AAG TGC AAT GCA GGA AG-3′ (forward) and 5′-GCA TCT GTT GAA CTT TAC GTC GG-3′ (reverse); *β-Actin*, 5′-TCC CTG GAG AAG AGC TAC G-3′ (forward) and 5′-GTA GTT TCG TGG ATG CCA CA-3′ (reverse). Real-time qRCR was conducted using SYBR Green dye in an M×3000PTM Real Time PCR amplification system (Stratagene, TX, USA). qPCR mixtures contained 2 µl cDNA (synthesized as described above), 12.5 µl SYBR Green Master mix (TaKaRa), and 0.5 µl of each upstream and downstream primer in a total volume of 25 µl. The amplification conditions were 95°C for 30 s, followed by 30 cycles of 95°C for 5 s, 60°C for 15 s, and 72°C for 10 s. Relative expression levels of *PROX1* were normalized to the geometric mean of *β-Actin* (internal control). The data were analyzed using the comparative threshold cycle (2^−ΔCT^, −ΔCT = CT_PROX1_−CT_β-Actin_) method.

### Immunohistochemistry

Sections (5 µm thick) were deparaffinized in xylene and rehydrated using a graded alcohol series. For antigen retrieval, the sections were heated in EDTA buffer (pH 9.0) for 20 min at 100°C. The tissue sections were then treated with 0.3% H_2_O_2_ for 5 min to block endogenous peroxidase activity and subsequently rinsed three times (2 min each) with phosphate-buffered saline (PBS). Rabbit anti-PROX1 antibody (Proteintech Group, CHI, USA) was used to detect PROX1 expression. The antibody was diluted 1∶100 in Tris-NaCl-blocking buffer (TNB) and incubated with the samples overnight at 4°C. After rinsing with PBS, tissue sections were incubated for 30 min with horseradish peroxidase (HRP)-conjugated mouse anti-rabbit secondary antibody. The slides were washed with PBS again, and incubated with the chromogenic substrate 3,3′-diaminobenzidine (DAB) to visualize the reaction. Finally, the sections were counterstained with hematoxylin, dehydrated, and mounted in Diatex. For negative immunostaining controls, the primary antibody was omitted. A hepatocellular carcinoma specimen was used as a positive control.

PROX1 expression was evaluated by two investigators blinded to clinicopathological information about the patients. Sections were considered to be positive when tumor cells showed cytoplasmic or nuclear PROX1 expression. Each tumor was given a score, obtained by multiplying the percentage of stained cells (0, 0%; 1, less than 25%; 2, 25–50%; 3, more than 50%) by the staining intensity (0, no staining; 1, weak staining; 2, moderate staining; 3, strong staining). Total scores of 0–3 were designated low expression, and total scores of 4–9 were designated high expression.

### Western blot analysis

Total protein of tissues and cells was obtained using RIPA lysis buffer (150 mM sodium chloride, 1% Triton X-100, 0.5% sodium deoxycholate, 0.1% sodium dodecyl sulphate (SDS), 50 mM Tris (pH 8.0)) containing a mixture of proteinase inhibitors. Protein concentration was determined using BCA protein assay reagent (Keygen, Nanjing, China). Equivalent amounts of proteins were separated by sodium dodecyl sulfate-polyacrylamide gel electrophoresis (SDS-PAGE) and then transferred onto nitrocellulose membranes (Invitrogen). After blocking in Tris-buffered saline (TBS) containing 5% fat-free milk, the membranes were incubated with primary anti-PROX1 (Upstate, NY, USA), anti-E-cadherin and anti-vimentin (Santa Cruz Biotechnologies, Santa Cruz, CA, USA) antibodies at 4°C overnight, and then incubated with HRP-conjugated secondary antibody at room temperature for 2 h. A β-Actin antibody (Sigma, St. Louis, MO, USA) was used as a control for equal loading. Blots were developed using an enhanced chemiluminescence (ECL) system (Pierce, Rockford, IL, USA), and signals were detected on X-ray film.

### Cell culture

The human RCC cell lines 786-O, 769-P, ACHN and OS-RC-2, and the human renal proximal tubular epithelial cell line HKC were obtained from the Cell Bank of the Chinese Academy of Science (Shanghai, China). 786-O, 769-P and OS-RC-2 cells were cultured in RPMI-1640 (Gibco, Gran Island, NY, USA); ACHN and HKC cells were maintained in Dulbecco's modified Eagle's medium (Gibco). All media were supplemented with 10% (v/v) fetal bovine serum (FBS; Gibco), penicillin (100 U/ml), and streptomycin (0.1 mg/ml), and cells were cultured in a humidified atmosphere of 5% CO_2_ and 95% air at 37°C.

### Plasmids and lentiviruses

Plasmid constructs were prepared as described previously [16].

Expression plasmids were transfected into cells using Lipofectamine 2000 (Invitrogen) according to the manufacturer's instructions. Recombinant lentiviruses were packaged by co-transfecting HEK293T cells with pLKO.1- or pWPI.1-based plasmids with the helper plasmids, pSPAX2 (Addgene plasmid 12260) and pMD2.G (Addgene plasmid 12259). Supernatants from co-transfections were used for infection of cultured cells. 786-O cells were infected with a *PROX1*-expressing lentivirus (Lenti-PROX1), and ACHN cells were infected with lentiviruses expressing small interfering RNA (siRNA) against *PROX1* (Lenti-si259, Lenti-si1646) or scrambled control siRNA (Lenti-siSCR). After 24 h, the infection medium was replaced with the cell-appropriate growth medium. Infected cells were passaged after ∼48–72 h.

### Cell proliferation assay

Cell proliferation was measured using a CCK-8 kit (Dojindo, kumamoto, Japan). A total of 1×10^3^ cells infected with lentiviruses were seeded into 96-well plates in 100 µl of medium containing 10% FBS and incubated at 37°C in 5% CO_2_. After 24, 48, 72 and 96 h, the medium was replaced with 100 µl of fresh medium and 10 µl CCK-8 solution was added to each well. Cells were then incubated for 2 h at 37°C in 5% CO_2_, after which absorbance at 450 nm was measured using a microplate reader (Molecular Devices, CA, USA). Each experiment was performed in triplicate and repeated in quadruplicate for each condition.

### Colony-formation assay

786-O and ACHN cells infected with lentiviruses were seeded separately in six-well plates at a density of 1×10^2^ cells/well. After incubation at 37°C for ∼10–14 d, cells were washed twice with PBS, stained with Giemsa solution (AppliChem, Darmstadt, German), and allowed to air dry at room temperature. The number of colonies containing more than 50 cells was microscopically counted, and colony-formation rate was calculated as the number of colonies/number of cells inoculated × 100%. Each experiment was performed in triplicate.

### Scratch-wound assay

For scratch-wound assays, 786-O and ACHN cells infected with lentiviruses were seeded in six-well plates and cultured until they reached 80–90% confluence. The cell layer in each well was scratched using a sterile 200 µl pipette tip to create a cleared line. After washing three times with PBS, cells were incubated under standard conditions, and migration into the scratched area was photographed (10-fold magnification) 0, 24, and 48 h after wounding.

### Statistical analyses

All statistical analyses were performed using SPSS 17.0 for Windows (SPSS, Inc.). Qualitative variables were compared using Person's χ^2^ test and Fisher's exact test, and quantitative variables were analyzed using Student's t test. Univariate analyses were calculated using the Kaplan-Meier method and were assessed using the log-rank test. Multivariate analysis was done using the Cox multivariate proportional hazards regression model. For *in vitro* experiments, individual culture dishes or wells were analyzed separately (i.e., no pooling of samples). Each experiment was repeated three times. Data are expressed as means ± standard deviation (SD). A one-way analysis of variance (ANOVA) was used for comparisons of means. A probability value of p<0.05 was considered to be statistically significant.

## Results

### Patient characteristics

Demographic and clinicopathological variables for the IHC cohort are summarized in Table 1. The mean age of the 115 patients was 60.9 ± 13.2 years; 64.4% of patients were male. Tumor stage was classified as T1/2 in 96 (83.5%) patients and T3/4 in 19 (16.5%) patients. Nuclear grade according to the Fuhrman classification was G1/2 in 92 (80%) patients and G3/4 in 23 (20%) patients. Lymph node involvement was present in 11 (9.6%) patients, and 13 (11.3%) patients had evidence of distant metastatic disease.

**Table 1:** Correlation between clinicopathological parameters and PROX1 expression.

Characteristics	All	PROX1 expression		*P*
		High	Low	
Patients, n (%)	115	39 (33.9)	76 (66.1)	
Age, n (%)				0.885
≤61	63 (54.8)	21 (53.8)	42 (55.3)	
>61	52 (45.2)	18 (46.2)	34 (44.7)	
Gender, n (%)				0.969
Male	74 (64.4)	25 (64.1)	49 (64.5)	
Female	41 (35.6)	14 (35.9)	27 (35.5)	
Primary tumor size, n (%)				0.493
≤7 cm	98 (85.2)	32 (82.1)	66 (86.8)	
>7 cm	17 (14.8)	7 (17.9)	10 (13.2)	
T-stage				0.059
T1/2	96 (83.5)	29 (74.4)	67 (88.2)	
T3/4	19 (16.5)	10 (25.6)	9 (11.8)	
N-stage				0.012
N0	104 (90.4)	31 (79.5)	73 (96.1)	
N1	11 (9.6)	8 (20.5)	3 (3.9)	
M-stage				0.955
M0	102 (88.7)	33 (84.6)	69 (90.8)	
M1	13 (11.3)	6 (15.4)	7 (9.2)	
Nuclear grade				<0.001
G1/2	92 (80.0)	20 (51.3)	72 (94.7)	
G3/4	23 (20.0)	19 (48.7)	4 (5.3)	

### PROX1 expression in RCC

**Figure 1:**
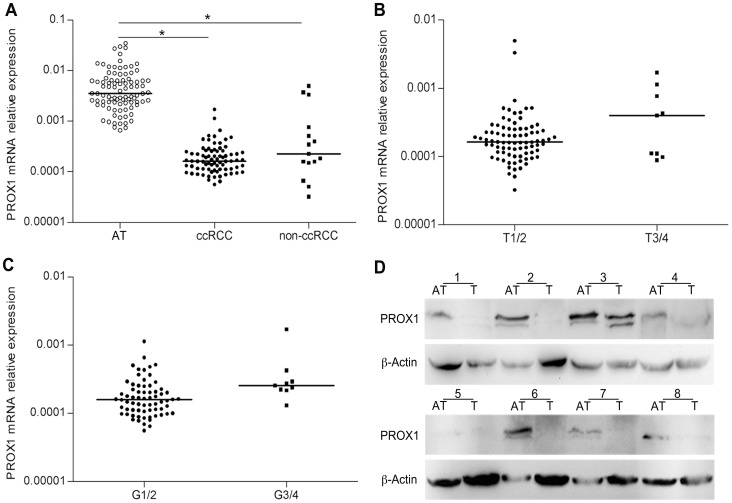
Real-time qPCR and Western blot analyses of *PROX1* expression in RCC and corresponding adjacent tissues. The relative expression of *PROX1* mRNA was significantly lower in both ccRCC and non-ccRCC tissue than in adjacent normal tissue (a). *PROX1* mRNA trended higher (though not significantly) in T3/4 and G3/4 RCC specimens compared with T1/2 and G1/2 specimens, respectively. (b and c). PROX1 protein expression levels were clearly reduced in 6 of 8 RCC specimens compared with adjacent normal tissue (d). Bars, median relative expression levels. *P<0.001.

Expression of *PROX1* mRNA was first assessed in 92 RCC specimens (77 ccRCC, 6 papillary RCC, 6 chromphobe RCC, 2 unclassified RCC, and 1 multilocular cystic RCC) and paired adjacent normal tissue. As shown in Figure 1a, *PROX1* expression was significantly reduced in both ccRCC and non-ccRCC tissues compared with matched adjacent tissue (both *P*<0.001). Unexpectedly, after stratifying by stage and grade, expression of *PROX1* mRNA trended higher in T3/4 and G3/4 RCC tissues compared with T1/2 and G1/2 tissues, respectively, although this difference did not reach statistical significance (Figure 1b and 1c). In addition, Western blot analysis showed that PROX1 protein was down-regulated in 6 of 8 RCC tissues compared with paired normal tissues (Figure 1d).

**Figure 2:**
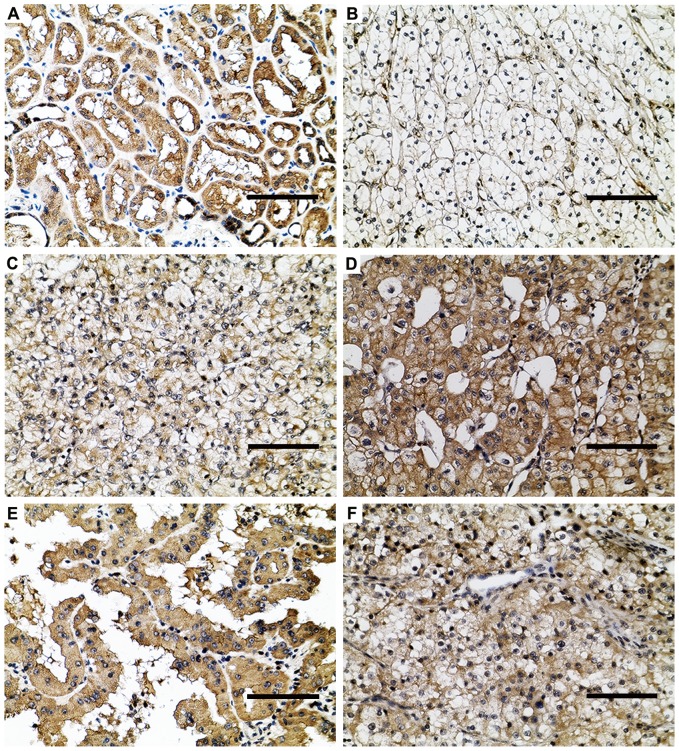
Representative immunostaining for PROX1 in RCC and adjacent normal tissues. Renal tubular epithelial cells showed intense cytoplasmic PROX1 expression (a). RCC tissues showed variable PROX expression; ccRCC showed low (b), moderate (c), or high (d) PROX1 staining. Papillary (e) and chromophobe (f) RCC samples showed high PROX1 expression. Scale bars, 100 µm.

To extend our observations, we tested PROX1 protein expression in paraffin-embedded RCC sections. Representative immunohistochemical staining results are shown in Figure 2. Specific staining for PROX1 was detected mainly in the cytoplasm in adjacent normal tissue; however, both cytoplasm and nuclei were PROX1 positive in tumor cells. Renal tubules in adjacent normal tissue showed intense PROX1 expression in 52 (89.7%) RCC patients; however, RCC tissues showed variable PROX1 expression levels. As summarized in Table 1, a total of 39 of 115 immunostained RCC specimens (33.9%) showed high expression and 76 (66.1%) showed low expression. The expression of PROX1 was clearly decreased in RCC compared with adjacent normal tissue (*P*<0.001). When stratified by tumor type, 30 (28.8%) ccRCC, 6 (85.7%) papillary RCC and 3 (75%) chromophobe RCC samples showed high PROX1 expression.

### Correlation of PROX1 expression and clinicopathological parameters in RCC

The relationships between PROX1 protein expression and clinicopathological parameters of RCC are summarized in Table 1. When specimens were stratified according to clinicopathological factors, PROX1 expression was found to be significantly related to tumor nuclear grade (*P*<0.001) and tumor N stage (*P* = 0.012). Similar to *PROX1* mRNA expression, PROX1 protein expression was also higher in T3/4 and G3/4 RCC specimens than in T1/2 and G1/2 specimens, respectively. Taken together, these findings indicate that PROX1 expression is associated with tumor differentiation and invasion, which are correlated with tumor progression.

### The prognostic significance of PROX1 expression in RCC

**Figure 3:**
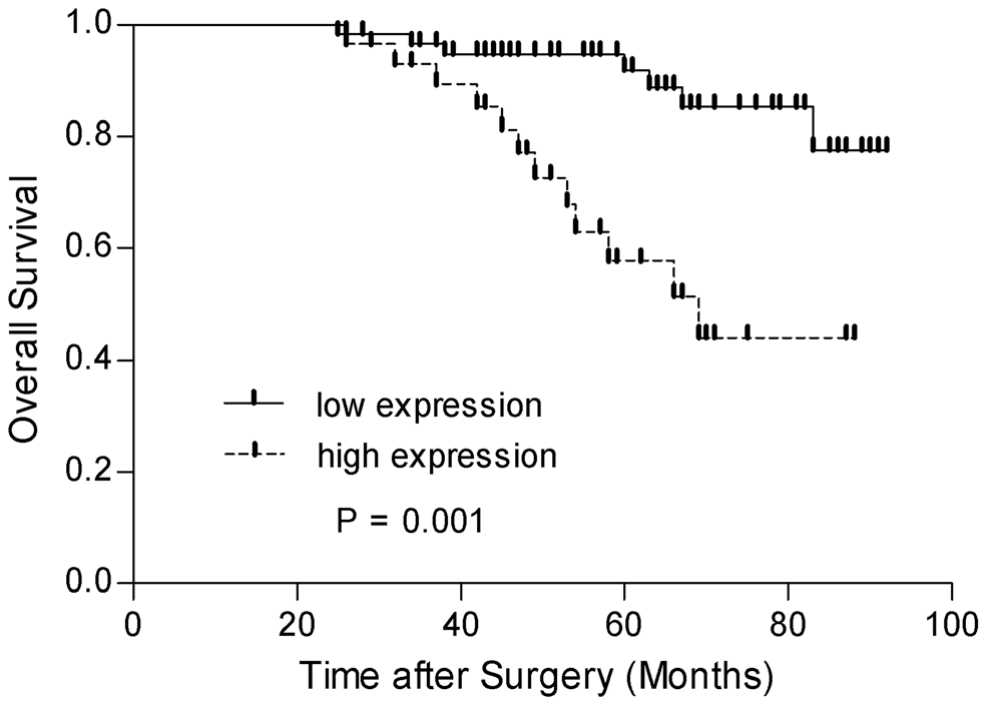
Kaplan-Meier curves of RCC patient OS according to PROX1 expression. OS is decreased in patients with high PROX1 expression compared with those with low levels of PROX1 expression (*P* = 0.001, Log-Rank test).

The follow-up time for the entire IHC cohort ranged from 25 to 92 months (median, 59). The association between PROX1 protein expression and overall survival (OS) was evaluated using a Kaplan-Meier survival analysis with log-rank statistic. As shown in Figure 3, OS was significantly decreased in the high PROX1 expression group compared to the low PROX1 expression group (*P* = 0.001).

Using the Cox proportional hazard model, we tested the independent predictive value of PROX1 expression as well as other clinicopathological parameters, including age, gender, tumor diameter, T-stage, N-stage, M-stage, and nuclear grade. As shown in Table 2, age (*P* = 0.002), tumor size (*P* = 0.013), T-stage (*P*<0.001), N-stage (*P* = 0.004), M-stage (*P*<0.001), nuclear grade (*P* = 0.003), and PROX1 expression (*P* = 0.001) were significantly associated with a higher risk of death. If adjusted in the multivariable model, age (*P* = 0.018), T-stage (*P* = 0.001), N-stage (*P* = 0.037), M-stage (*P*<0.001) and PROX1 expression (*P* = 0.005) were also significantly associated with OS.

**Table 2:** Univariate and multivariate analyses of prognostic factors associated with overall survival in RCC.

Variables	Overall survival			
	Univariate	Multivariate		
	*P*	HR	95% CI	*P*
Age, years (>61 vs. ≤61)	0.002	4.056	1.277–12.881	0.018
Gender (male vs. female)	0.674			NA
Tumor size (>7 cm vs. ≤7 cm)	0.013			NS
T-stage (T3/4 vs. T1/2)	<0.001	5.31	1.913–14.733	0.001
N-stage (N1 vs. N0)	0.004	3.932	1.048–14.269	0.037
M-stage (M1 vs. M0)	<0.001	9.411	2.882–30.735	<0.001
Nuclear grade (G3/4 vs. G1/2)	0.003			NS
PROX1 expression (high vs. low)	0.001	5.015	1.638–15.353	0.005

Univariate analysis was performed using the Kaplan-Meier method (log-rank test). Multivariate analysis was performed using the Cox multivariate proportional hazards regression model in a stepwise manner (backward, conditional) NA not adopted, NS not significant, HR hazard ratio, CI confidence interval.

### Effects of PROX1 overexpression and depletion on cell proliferation and colony formation *in vitro*

**Figure 4:**
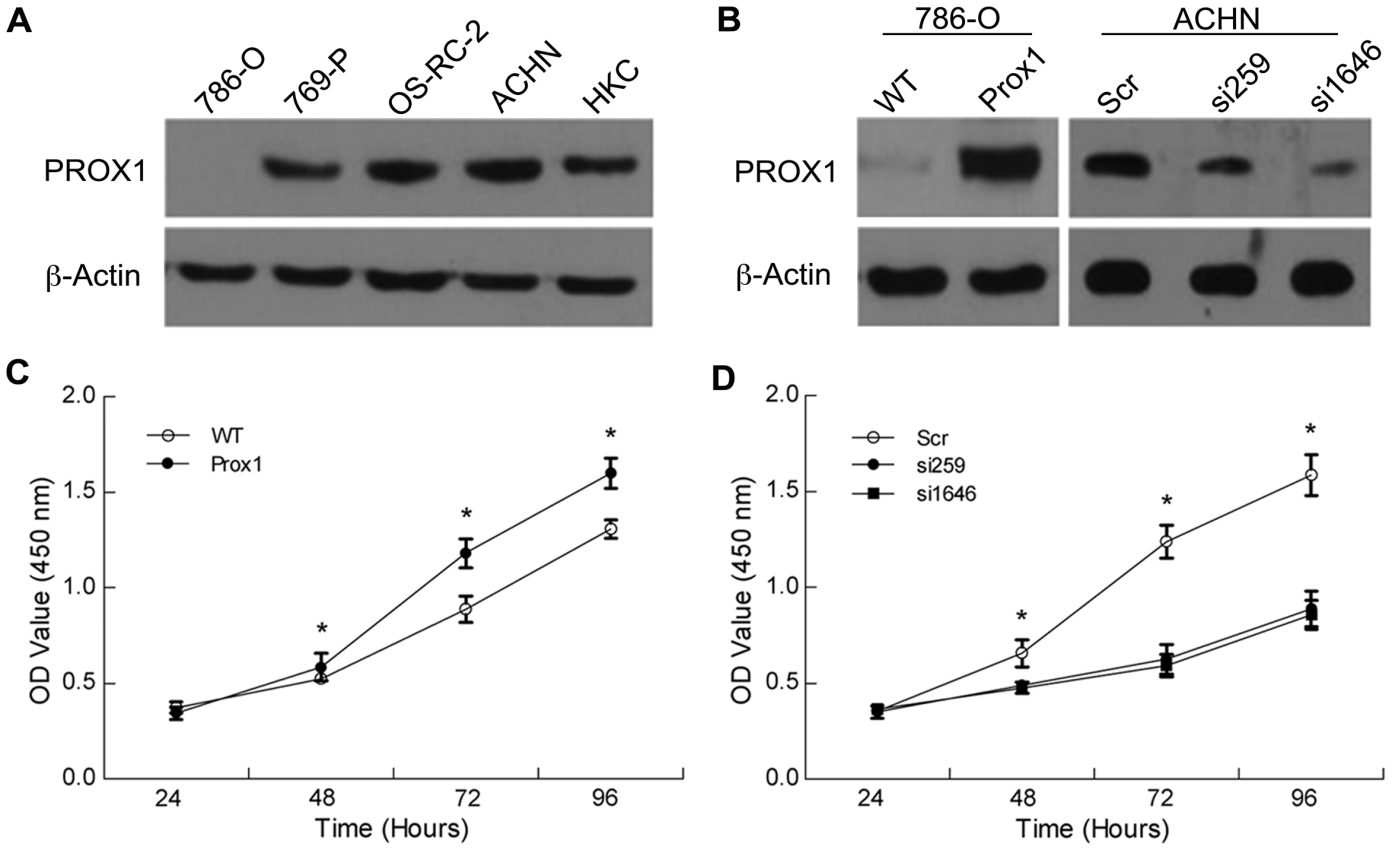
PROX1 protein expression in RCC cells and the effect of PROX1 on cell proliferation. (a) PROX1 expression in wild-type RCC cells (786-O, 769-P, OS-RC-2, and ACHN) and human renal proximal tubular epithelial cells (HKC). (b) PROX1 expression in RCC cells infected with lentiviruses. (c) Overexpression of *PROX1* in 786-O cells significantly increased cell proliferation rate. (b) Knockdown of endogenous *PROX1* expression in ACHN cells dramatically reduced cell proliferation rate. *P<0.001.

We evaluated the expression of PROX1 in renal cell carcinoma cell lines, including 786-O, 769-P, OS-RC-2 and ACHN, as well as the human renal proximal tubular epithelial cell line HKC. As shown in Figure 4a, 786-O cells were nearly negative for PROX1 expression; however, the three other cancer cell lines as well as HKC cell clearly expressed PROX1. On the basis of these findings, we used lentivirus-mediated overexpression of *PROX1* in 786-O cells and siRNA-mediated knockdown of *PROX1* expression in ACHN cell to examine the potential effects of PROX1 on the behavior of RCC cells.

PROX1 protein expression was markedly enhanced in 786-O cells infected with Lenti-PROX1 compared with wild-type cells, whereas PROX1 protein expression was effectively knocked down in ACHN cells infected with Lenti-si259 or Lenti-si1646, targeting *PROX1*, compared with those infected with Lenti-siSCR, expressing a scrambled control siRNA (Figure 4b).

**Figure 5:**
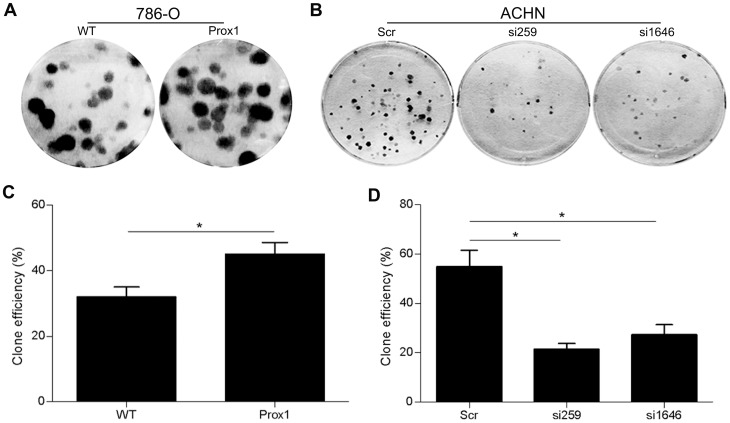
PROX1 enhances colony formation *in vitro*. (a, c) Representative picture of plates from colony-formation assays using RCC cells infected with lentiviruses. (b, d) Quantification of colony-formation assays. *P<0.001.

After infecting 786-O cells with Lenti-PROX1 and ACHN cells with Lenti-si259, Lenti-si1646 or Lenti-siSCR, as indicated above, we examined cell proliferation using CCK-8 assays. Overexpression of *PROX1* enhanced the growth of 786-O cells (Figure 4c), whereas down-regulation of *PROX1* exerted the opposite effect in ACHN cells (Figure 4d). This discrepancy in growth behavior between *PROX1*-overexpressing and *PROX1*-knockdown cells increased over time. To extend this analysis, we performed colony-formation assays. The results of these assays confirmed the enhanced proliferative potential of *PROX1*-overexpressing 786-O cells (Figure 5a and 5c) and reduced proliferative potential of *PROX1*-silenced ACHN cells (Figure 5b and 5d). Collectively, the results of CCK-8 and colony-formation assays suggest that PROX1 expression influences the growth and proliferation of RCC cells *in vitro*.

### Effects of PROX1 overexpression and depletion on cell migration and E-cadherin and vimentin expression *in vitro*

**Figure 6:**
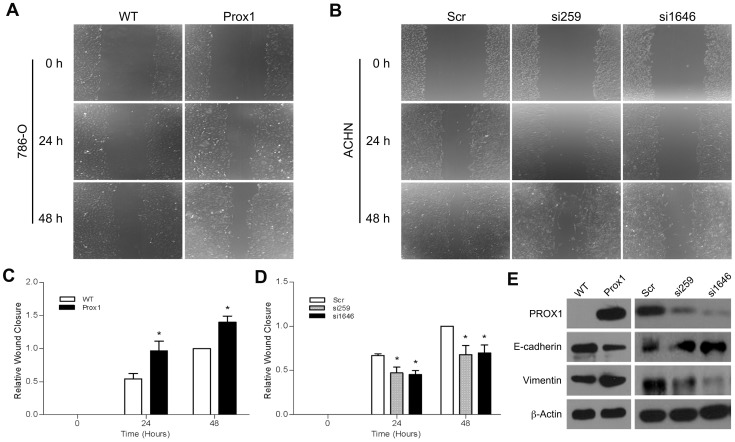
PROX1 enhances cell wound closure *in vitro* and regulates E-cadherin and vimentin protein expression. (a) 786-O cells were infected with Lenti-PROX1 for 72 h in six-well plates. The cell layer was scratched in each well to create a cleared line. The cells were then incubated in fresh media. (b) The same experiment was performed in ACHN cells infected with Lenti-siSCR, Lenti-si259, or Lenti-si1646. (c,d) The wound width was calculated using Image-Pro Plus software, and the relative wound closure was calculated, setting wound closure of the control condition at 48 h as 1. (e) Western blot analysis of PROX1, E-cadherin, and vimentin protein expression in 786-O and ACHN cells infected with lentiviruses for 72 h. *P<0.001.

We further examined the cytological effect of PROX1 on the migration ability of RCC cells using a scratch-healing assay. *PROX1*-overexpressing 786-O cells largely sealed the wound 24 h after scratching (Figure 6a and 6c); in contrast, *PROX1*-silenced ACHN cells only partially sealed the wound at this time point (Figure 6b and 6d). Furthermore, overexpression of *PROX1* correlated with decreased E-cadherin expression and increased vimentin expression in 786-O cells. Conversely, reduced PROX1 correlated with *E-cadherin* overexpression and reduced vimentin expression in ACHN cell (Figure 6e).

## Discussion

The present study represents the first examination of the tumorigenic and prognostic significance of altered PROX1 protein expression in RCC patients. In our initial studies, we found that both *PROX1* mRNA and protein expression were clearly reduced in RCC tissues compared with adjacent normal tissues. Unexpectedly, however, the aberrant expression of PROX1 was positively correlated with advanced disease stages and metastasis, and negatively correlated with patients' OS. Consistent with clinical findings, experiments on RCC cell lines demonstrated that, on the one hand, PROX1 overexpression dramatically enhanced proliferation and migration of RCC cells *in vitro*, and on the other hand, PROX1 depletion significantly inhibited proliferation and migration of RCC cells *in vitro*. Collectively, these results indicate a critical role for PROX1 in driving disease progression and spread of RCCs.

Recent studies have demonstrated that higher PROX1 protein expression in gliomas is indicative of a more aggressive phenotype [17]. An analysis of a large patient population revealed that high PROX1 expression was associated with poorly differentiated colorectal cancer and less favorable patient outcomes [18]. We also previously documented that high PROX1 protein expression in primary hepatocellular carcinoma (HCC) tissues was correlated with worse patient survival, in addition, PROX1 promoted HCC cell metastasis *in vitro* and *in vivo* [16]. In contrast, *PROX1* mRNA expression was markedly decreased in lymphoid malignancies and breast carcinoma tissues [19], [20]. Although *PROX1* mRNA was slightly down-regulated in pancreatic carcinomas, immunofluorescence revealed variable PROX1 protein expression in pancreatic carcinomas [12]. Another study of liver tumor found that *PROX1* mRNA expression was highly variable among samples of normal, cirrhotic, HCC and cholangiocellular carcinoma (CCC) human liver specimens, specifically showing that expression was decreased in HCC and CCC liver samples relative to normal controls, and was stably elevated in HCC cell lines [21]. As is the case during development, the role of PROX1 in a variety of human cancer types also appears to be tissue dependent and reflect both oncogenic and tumor-suppressive potential. In the present communication, we demonstrated that, although PROX1 expression in RCC tissue was lower than that in adjacent normal tissue, high expression was correlated with poor patient survival. These results imply that down-regulation of PROX1 expression may promote the early stage of RCC progression. We speculate that, by eliminating PROX1-mediated regulation of cell differentiation, down-regulation of PROX1 may be an important phenomenon in the progression from normal to precancerous cells or in situ establishment of early cancer status. However, up-regulation of PROX1 expression during the transition from localized to advanced cancer stages may imply altered promotion of cell proliferative and invasive functions at this stage of disease progression, strengthening the opinion that PROX1 exerts its function in a context-dependent manner. However, a clearer understanding of the underlying mechanisms through which PROX1 acts in different stages of tumor development will require further investigation.

One of key processes in the development of metastatic disease is the loss of cellular adhesion [22]. E-cadherin, a member of the cadherin family of adhesion molecules, is responsible for maintaining interactions of epithelial cells [23]. Our research on HCC has detected that the expression of E-cadherin was down-regulated in *PROX1*-overexpressing cell and up-regulated in *PROX1*-knockdown cells, and the expression of vimentin was reversely related with the change of E-cadherin [16]. In accord with our findings, Lu and colleagues found that forced expression of *PROX1* in colon cancer cells also down-regulated E-cadherin expression and attenuated cell adhesion; conversely, knockdown of *PROX1* restored E-cadherin expression and reduced invasiveness [24]. In the case of squamous cell carcinoma, E-cadherin-mediated cell-cell adhesion was found to induce epidermal growth factor receptor (EGFR) activation, which triggers the ERK/MAPK signaling module and further blocks down-regulation of the anti-apoptotic protein Bcl-2, promoting tumor cell survival [25]. Given the role of PROX1 in down-regulating the E-cadherin tumor-suppressor protein, it is likely that E-cadherin is involved in PROX1-stimulated proliferation and migration of tumor cells.

It is well known that vimentin is a prominent member of the intermediate filament family of proteins whose overexpression in cancer correlates well with increased tumor growth, invasion, and poor prognosis [26]. In prostate cancer, abrogating the expression of vimentin significantly decreases tumor cell invasiveness, an effect that has been attributed to its ability to regulate the E-cadherin/β-catenin complex via c-Src regulation [27]. Utilizing oligonucleotide microarrays and gene set enrichment analyses, Chen D et al. showed that down-regulation of E-cadherin and low vimentin levels were correlated with RCC metastasis and poor prognosis, providing strong evidence that epithelial-mesenchymal transition (EMT) occurs in RCC [28]. Our *in vitro* experiments revealed that *PROX1*-overexpressing 786-O cells showed a more aggressive phenotype in association with reduced E-cadherin and enhanced vimentin expression, whereas *PROX1* down-regulated ACHN cells showed a less aggressive phenotype accompanied by enhanced E-cadherin and reduced vimentin expression. Given that both E-cadherin and vimentin are generally regarded as critical markers of EMT, these data indicate that expression of PROX1 may contribute to the development of an invasive phenotype in conjunction with E-cadherin and vimentin during the process of EMT in RCC.

To date, no specific biomarker of renal cell carcinoma has been developed for use in clinical diagnosis and prediction of prognosis. Many of the oncogenes and tumor-suppressor genes whose mutation leads to dysregulation of cellular pathways in RCC remain to be elucidated. To our knowledge, this is the first study to evaluate the possibility of using PROX1 as a potential clinical indicator of disease progression, as well as a prognostic marker for patient survival in RCC. Although PROX1 does not appear to be a specific RCC marker, its significance in predicting tumor progression and prognosis suggest that it could benefit RCC patients.

In conclusion, our findings revealed that the expression levels of PROX1 in RCC tissues are divergent and lower on average than those in adjacent normal tissues. Unexpectedly, elevated PROX1 expression in RCC was found to be associated with a more malignant phenotype and poorer prognosis. In agreement with clinical findings, *in vitro* experiment confirmed that PROX1 conferred aggressive characteristics on RCC cells. Additionally, PROX1 may exert its function by interacting with E-cadherin and vimentin during EMT; however, further study will be required to elucidate the role of E-cadherin and vimentin in PROX1-mediated RCC progression.
